# Cardiac Screening of Young Athletes: a Practical Approach to Sudden Cardiac Death Prevention

**DOI:** 10.1007/s11936-018-0681-4

**Published:** 2018-08-28

**Authors:** Harshil Dhutia, Hamish MacLachlan

**Affiliations:** 1 Cardiovascular Sciences Research Centre, St George’s University of London, London, UK; 2 Department of Cardiology, Glenfield Hospital, Leicester, UK

**Keywords:** Pre-participation cardiovascular screening, ECG, Sudden cardiac death, Sports cardiology, Cardiomyopathy, Athlete

## Abstract

**Purpose of review:**

We aim to report on the current status of cardiovascular screening of athletes worldwide and review the up-to-date evidence for its efficacy in reducing sudden cardiac death in young athletes.

**Recent findings:**

A large proportion of sudden cardiac death in young individuals and athletes occurs during rest with sudden arrhythmic death syndrome being recognised as the leading cause. The international recommendations for ECG interpretation have reduced the false-positive ECG rate to 3% and reduced the cost of screening by 25% without compromising the sensitivity to identify serious disease. There are some quality control issues that have been recently identified including the necessity for further training to guide physicians involved in screening young athletes.

**Summary:**

Improvements in our understanding of young sudden cardiac death and ECG interpretation guideline modification to further differentiate physiological ECG patterns from those that may represent underlying disease have significantly improved the efficacy of screening to levels that may make screening more attractive and feasible to sporting organisations as a complementary strategy to increased availability of automated external defibrillators to reduce the overall burden of young sudden cardiac death.

## Introduction

The sudden death of a young individual is a tragic and highly emotional event. Apart from the devastation within a family unit, the sudden nature of the event and the loss of decades of life have a lasting impact on friends, peers, and both the lay and medical communities. Deaths are usually attributable to hereditary or congenital abnormalities affecting the cardiac structure or the electrical system of the heart. Such cases galvanise discussion between physicians, health authorities and the lay community with an emphasis on improving understanding of the conditions predisposing to sudden cardiac death (SCD) and development of effective preventative strategies.

Much of the data on SCD in young individuals (aged under 35 years) is derived from studies of highly trained young competitive athletes. Deaths in this cohort, whilst rare, are often high profile and are afforded significant visibility. Identification of athletes at risk of SCD has become an important focus of the medical community on the premise that the majority of responsible diseases can be detected during life, and acceptable interventions such as lifestyle, pharmacological therapy, radiofrequency ablation of accessory pathways or implantation of a cardioverter defibrillator are available to reduce the risk of SCD.

Pre-participation cardiovascular screening (PPS) of young athletes has been endorsed by learned scientific organisations bodies and sporting governing bodies including the European Society of Cardiology (ESC), the American Heart Association (AHA), International Olympic Committee (IOC) and Fédération Internationale de Football Association (FIFA) [1–4]. However, screening of athletes is still not universally accepted due to concerns primarily related to the lack of randomised control evidence for its efficacy, reliability issue and cost. This review article will aim to address the current status of cardiovascular screening of athletes worldwide and review the up-to-date evidence for its efficacy in reducing SCD in young athletes.

## Sudden cardiac death in athletes: the magnitude of the problem

There is widespread agreement that SCD is the leading medical cause of death in athletes [5, 6]. Current estimates of the incidence of SCD in athletes vary widely from almost one in a million to 1:23,000 athletes per year, whilst some subpopulations of athletes are reported at even higher risk with an incidence of 1 in 3000 [5, 7••]. Accurate calculation of the incidence of SCD in athletes requires a precise numerator (number of deaths per year) and an exact denominator (number of athlete participants per year) in the population studied. Variability in either of these accounts for unreliable estimates of incidence. It is challenging to compare studies with heterogeneous case identification methods that originate from different geographic regions. Passive collection methods using retrospective review of media reports, electronic databases and insurance claims are limited by ascertainment and selection bias which underestimate calculations of incidence. Mandatory reporting systems of athlete’s deaths within good population demographics offer the most reliable method of case identification although very few currently exist. Furthermore, the inclusion of all cardiac events (including survivors of sudden cardiac arrest (SCA)) versus only those resulting in death and the population examined can impact on the incidence estimate. The increasing number of athletes surviving SCA may provide the misconception of drastically falling rates of SCD. Primary preventative programs target both SCA and SCD; therefore, it is prudent to incorporate survivors of SCA in estimates of incidence. Harmon et al. performed a comprehensive review of studies that have examined the incidence of SCD in athletes. The objective of this review was to assess the methodological strengths and weaknesses used to arrive at estimates, compare studies with estimates of similar populations and arrive at an approximation of incidence based on the based available evidence. The incidence of SCD across all 28 studies varied from 1:3000 to 1:917,000; however, studies with higher methodological quality yielded a higher estimate of incidence ranging from 1:40,000 to 1:80,000 [7••]. Overall, an incidence of 1:50,000 per year is generally accepted as the incidence of SCD in young athletes. Whilst this rate is fortunately relatively low, hereditary and congenital abnormalities of the heart are the leading causes of non-accidental death in young athletes [5, 6].

## Demographics of sudden cardiac death in young athletes

Certain populations of sportspeople also seem to be at greater risk. There is a significant male predominance in SCD amongst young athletes. Data from the National Centre for Catastrophic Sports Injury Research in high school and college athletes reported a 5-fold higher incidence of SCD in male compared to female athletes [8]. In the Veneto region of Italy, where over 110,000 athletes were evaluated over a 25-year follow-up period, incidence rates of SCD were 2.6 per 100,000/year in male athletes and 1.1 per 100,000/year in their female counterparts [9]. Similarly, in a study of National Collegiate Athletic Association (NCAA) athletes, male collegiate athletes were at 3-fold higher risk of SCD compared to female athletes [5, 10]. Several factors are implicated in this sex difference including a higher prevalence of premature coronary artery disease in young male athletes and lower participation rates among female athletes particularly at the elite level, although this trend is changing.

There is limited reported data on incidence of SCD in young individuals of African/Afro-Caribbean (black) ethnicity within the general population in spite of the explosion in the number of athletes of this ethnicity competing at the elite level over the last three decades. Recent studies of the incidence of SCD in NCAA athletes reported that the incidence of SCD in black athletes as 5.6/100000 per year, 3-fold higher compared to white athletes [5, 10]. SCD occurs more frequently in certain sporting disciplines. In the USA, basketball and American football have the greatest incidence, whereas in Europe, soccer predominates [9, 10]. However, there is the potential for data bias due to higher participation rates in these sporting disciplines. Although there is a high proportion of participation by black athletes in sports such as basketball, there are no notable differences in rates of SCD between black and white collegiate basketball players. This suggests there may be an intrinsic element of the sport beyond the ethnic/racial make-up of the participants that predisposes to SCD. Extrapolation of these observations suggests that individuals participating in sports of high dynamic and low isometric intensity are at higher risk of SCD.

## Aetiology of sudden cardiac death in young athletes

Understanding both the aetiology and precipitating factors of SCD is paramount when devising preventative strategies such as PPS and widespread availability of automated external defibrillators (AEDs). SCD in young individuals is attributable to hereditary and congenital abnormalities of the heart (Table 1). Based on several independent international studies, the combined prevalence of these diseases in athletes is estimated at 0.3% [11]. Additionally, there are several important acquired causes during exercise-related SCD. Cardiomyopathy is the commonest cause of SCD in young athletes worldwide [6, 12, 13]. Specifically, HCM is the leading cause of SCD in the USA, whilst in Italy, arrythmogenic cardiomyopathy (AC) predominates [9]. Regional discrepancies between studies reporting HCM and AC as the commonest cause of SCD may be explained by genetic variation, ascertainment bias of identified cases, and different criteria for pathological diagnosis.

**Table 1:** Causes of sudden cardiac death (SCD) in young sportspeople

Congenital/genetic pathology	
Disease of the myocardium	Hypertrophic cardiomyopathy Arrhythmogenic ventricular cardiomyopathy Dilated cardiomyopathy
Coronary artery disease/anomalies	Congenital coronary artery anomalies Premature atheromatous coronary artery disease
Cardiac conduction tissue abnormalities	Wolff–Parkinson–White syndrome Right ventricular outflow tachycardia
Valvular heart disease and disorders of the aorta	Mitral valve prolapse Congenital aortic stenosis Marfan syndrome
Ion channelopathies	Congenital long QT syndrome Catecholaminergic polymorphic ventricular tachycardia Brugada syndrome
Acquired causes	
Infections (myocarditis)	
Drugs (cocaine, amphetamine)	
Electrolyte disturbances (hypokalemia or hyperkalemia)	
Hypothermia	
Hyperthermia	
Trauma (commotio cordis)	

Recent data from the USA, Australasia and the UK suggest that autopsy negative sudden unexplained death in athletes with presumed SCD may be more common than previously thought. Data from a specialist cardiology pathology centre in the UK in 357 athletes has shown that in up to 42% of cases, the heart is structurally normal, and when the toxicology screen is negative, these deaths are classified as sudden arrhythmic death syndrome (SADS) [14]. Such cases are often attributed to primary cardiac ion channel disorders such as the long QT syndrome (LQTS), Brugada syndrome, catecholaminergic polymorphic ventricular tachycardia (CPVT) or the congenital accessory pathways. An accurate diagnosis of SADS is of vital importance when considering a diagnosis of an inherited ion channelopathy or cardiomyopathy is made in up to 50% of families later evaluated [15]. The role of molecular autopsy has been promoted to help shed light of the aetiology of SCD in such cases even when autopsy is performed at specialised centers [16].

## Outcomes of mandatory screening programs in athletes

### The Italian experience

The most persuasive evidence supporting the theory that early identification of disease through ECG screening saves lives comes a large prospective Italian study of 42,386 competitive athletes aged 12–35 years with 26-year follow-up [17]. PPS is mandatory in Italy by law, with standard evaluation comprising of history, physical examination and resting 12-lead ECG. The study compared the incidence of SCD in athletes in the pre-screening era (1979–1982) and late screening eras (2003–2004). Fifty-five cases were identified over the course of the study. The study demonstrated a reduction in the incidence of SCD from 3.6/100,000 person-years to 0.4/100,000 person-years, representing a 90% reduction in mortality. The predominant reason for this reduction was a decrease in SCD due to cardiomyopathy, particularly AC which was a relatively novel entity during the pre-screening era.

### Israel and the USA

The success of the Italian PPS experience has not been replicated in other countries, with studies in Israel and the USA demonstrating no significant benefit from PPS in young athletes. Similar to Italy, a mandatory PPS program by law in Israel since 1997 requires competitive athletes aged 17–34 years to undergo PPS inclusive of the 12-lead ECG. As in the Italian study, the impact of screening on SCD risk has been estimated in an Israeli study comparing the incidence of events before and after implementation of screening [18]. Case identification was derived through media searches from 2 national newspapers on a daily basis. The study reported 24 SCD events with an average annual mortality incidence of 2.54 per 100,000/year in the 12 years prior to the implementation of nationwide screening and 2.66 per 100,000/year in the 12 years after (*p* = 0.88), consistent with screening having no apparent effect on SCD rates.

There have been no national screening programs in the USA similar to Italy and Israel. However, cardiovascular mortality rates have been estimated in Minnesota, where athletes are evaluated with history and physical examination alone [19]. Case identification was based mostly on a retrospective analysis of data provided by different sources, such as the Minnesota State High School League, news media information services, and internet search engines. Over a 23-year period, 22 deaths were identified. The investigators reported an incidence of SCD of < 1 per 100,000 years throughout the study.

The studies from Israel and USA suggest that PPS of competitive athletes has little effect on SCD mortality, thus contradicting the findings from Italy. Critics of the Italian data often cite that the reduced mortality cannot be equivocally attributed to ECG screening as the study was an observational cohort based investigation, and not a randomised controlled trial. However, it would be impossible to conduct a randomised control trial in Italy as screening of competitive athletes is mandatory by law. The Italian data is further strengthened by the prospective study design and case identification through systematic mandatory reporting system for juvenile sudden death with autopsies performed by specialist cardiovascular pathologists, equating to a more reliable denominator when calculating mortality rates. In Israel and the USA, data collection was retrospective and collected predominantly from media sources and catastrophic insurance claims leading to likely underestimate the true mortality rates in these populations. Moreover, both the Israel and USA studies estimated the true number of athletes participating in sports each year.

## Policy to screen young athletes

The AHA and ESC both advocate PPS of young athletes on ethical, medical and legal grounds. However, they differ in that the AHA recommends taking a thorough medical and family history along with a physical examination, whereas the ESC recommends routine addition of a 12-lead ECG in the initial screening stages [1, 20]. The American model is cheap and pragmatic but has poor sensitivity. In a systematic review/meta-analysis of 15 studies comparing screening strategies in 47,137 athletes, the sensitivity/specificity of ECG was 94%/93%, history 20%/94% and physical examination 9%/97% to identify cardiovascular disease associated with SCD [11]. These findings are not entirely surprising as most athletes are asymptomatic before SCD, and most diseases implicated in SCD during sports are not associated with physical signs [21]. A family history is often absent even in affected athletes, because diseases such as HCM and LQTS have low event rates; therefore, family members may not have presented with a sentinel event. In a seminal article, Maron et al. described the demographics of 134 young athletes with SCD [22]. Of 115 young athletes who died suddenly and who had had a standard pre-participation AHA medical evaluation, only 4 (3%) were suspected of having heart disease, and the abnormality responsible for the death was correctly identified in only 1 athlete (0.9%).

## ECG interpretation criteria in athletes

Intensive exercise training results in the development of a constellation of physiological alterations in autonomic tone, cardiac structure and cardiac function which may be represented by electrical anomalies on the ECG. On occasion, the patterns of electrical alterations related to athletic conditioning overlap with those observed in individuals with cardiomyopathy. Misinterpretation of these benign physiological ECG changes is not uncommon, particularly when performed by physicians without expertise in sports cardiology [23]. False-positive ECG findings can result in unnecessary, costly secondary investigations and disqualification from sport. The high false-positive ECG rates and impact on specificity are commonly cited as major limitations of the ECG as a screening test in young athletes [24].

**Fig. 1:**
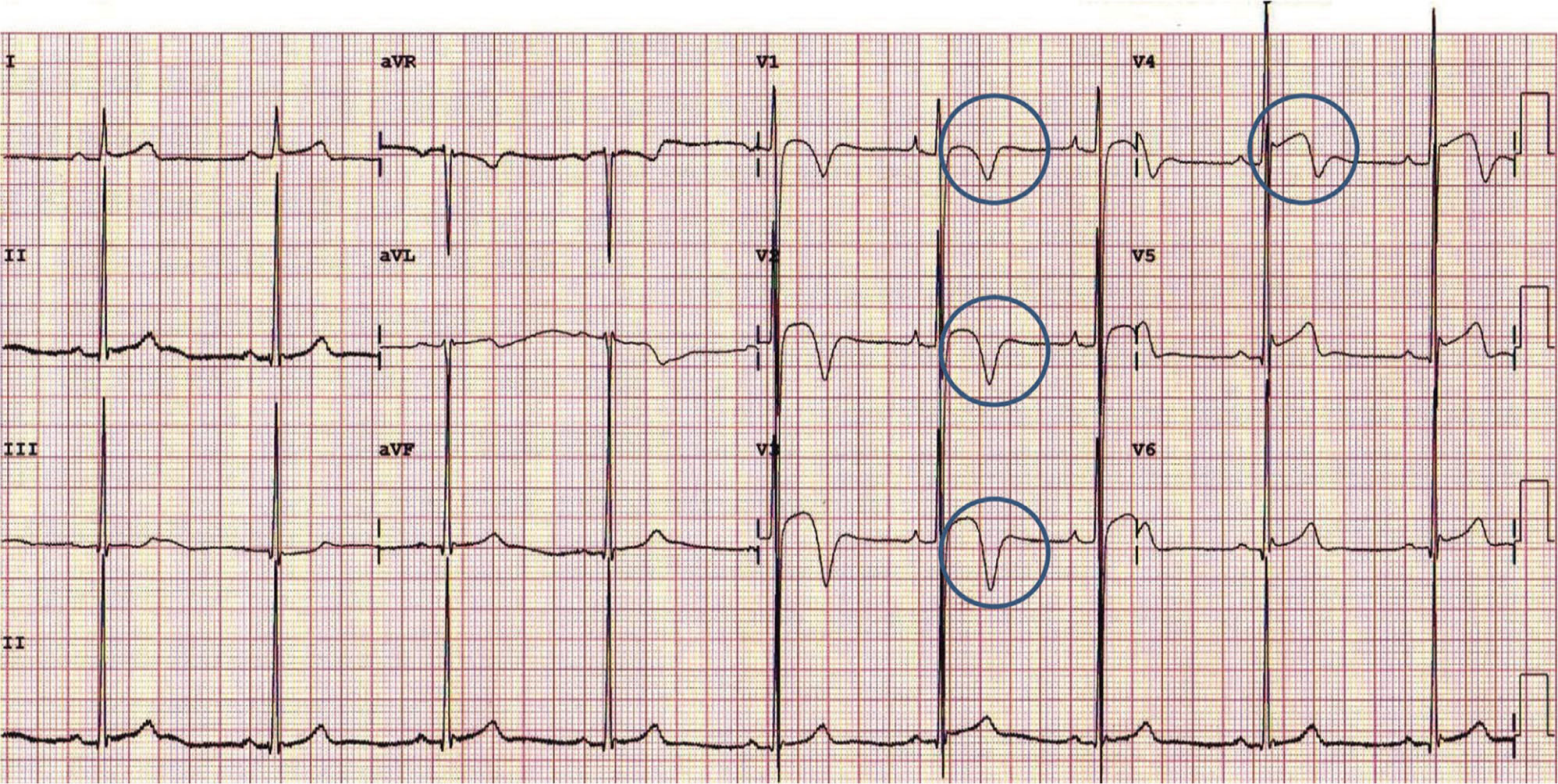
Electrocardiogram from a Black athlete demonstrating voltage criterion for left ventricular hypertrophy, J-point elevation, and convex ST segment elevation followed by T-wave inversion in V1 to V4 (circles) [34••].

The past three decades has seen a tremendous rise in black athletes competing at every level of sport. Data from pre-participation programs have informed our knowledge particularly of black athletes. It is now well established that as with white counterparts, black athletes reveal a high prevalence of benign, exercise-related ECG changes, particularly ST segment elevation, voltage criteria for LVH and early repolarisation [25–27]. It is also apparent that black athletes demonstrate a higher prevalence of T-wave inversion which would be deemed abnormal in white athletes and often associated with primary cardiomyopathies. Specifically, T-wave inversion preceded by J-point elevation and an ST segment with a convex morphology in the anterior leads V1-4 (Fig. 1) is present in nearly 13% of highly trained black athletes and is considered a benign phenomenon [26].

A lower prevalence of ECG abnormalities has consistently been reported in adult female athletes compared with male athletes. In an Italian study of an unselected cohort of 32,652 athletes undergoing PPS, females demonstrated an abnormal ECG prevalence of 9.6% vs. 12.4% in males (*p* = 0.0001) [28].

It is now recognised that athletes participating in certain sporting disciplines involving a greater combination of intensity and duration (i.e. endurance athletes) may have different frequencies of ECG changes than non-endurance athletes. In a study of 1007 male and 254 female elite adult athletes undergoing PPS, physiological ECG changes were more common in endurance athletes than non-endurance athletes (90.8% vs 86.0%, *p* = 0.04), as were multiple (≥ 2) training-related changes (78.9% vs 53.5%, *p* < 0.0001) [29]. ECG changes considered uncommon or pathological were seen in 18.1% of subjects and were twice as prevalent in endurance athletes compared with non-endurance athletes (29.9% vs 15.1%, *p* < 0.0001). RVH and deep right precordial T-wave inversion were three times as common in endurance athletes and were thought to be as a consequence of increased structural and/or electrical RV remodeling in this subgroup.

Reservations exist around creating unnecessary anxiety for young athletes and their families; falsely restricting an athlete from competitive sport can have significant financial, psychological and social implications. More concerning however is the potential for erroneous diagnosis resulting in a false reassurance for athletes harboring cardiovascular disease associated with SCD. To address the issue of high false-positive ECG rates, the last decade has seen several guidelines for ECG interpretation published to aid the physician in the interpretation of the athlete’s ECG by differentiating physiological ECG changes from those that may represent underlying disease. Specifically, the 2010 ESC recommendations, Seattle criteria and refined criteria were developed and sequentially improved specificity of ECG screening by reducing the false-positive rates from 22–25% to 5% by accounting for ECG changes that are physiological in athletes as well as the impact of ethnicity on the athlete’s ECG [30–33].

In 2017, the international recommendations were devised by a group of American and European experts with the overarching aim of unifying the recommendations for interpretation of the athlete’s ECG [34••]. These recommendations account for adolescent athletes, black ethnicity and some non-specific electrical anomalies, notably axis deviation and voltage criteria for atrial enlargement. These criteria (Table 2) have been validated in a single nationwide study of nearly 5000 young British athletes and have demonstrated further reduction in the proportion of athletes requiring further investigation to 3%, rates which are likely to be acceptable to the any screening program [35].

**Table 2:** International consensus standards for electrocardiographic interpretation in athletes

Normal	Borderline*	Abnormal
QRS voltage consistent for LVH or RVH	Left axis deviation	T waver inversion
Incomplete RBBB	Left atrial enlargement	ST segment depression
Early repolarisation/ST segment elevation	Right axis deviation	Pathologic Q waves
ST segment elevation and TWI V1–V4 in Black athletes	Right atrial enlargement	Complete LBBB
TWI V1–V3 age < 16 years old	Complete RBBB	QRS ≥ 140 ms duration
Sinus bradycardia or arrhythmia		Epsilon wave
Ectopic atrial or junctional rhythm		Prolonged QT interval
1st degree AV block		Ventricular pre-excitation
Mobitz type I 2nd degree AV block		Brugada Type 1 pattern
		Profound sinus bradycardia < 30 bpm
		PR interval ≥ 400 ms
		Mobitz type II 2nd degree AV block
		3rd degree AV block
		≥ 2 PVCs
		Atrial tachyarrhythmias
		Ventricular tachyarrhythmias

*AV* atrioventricular block, *LBBB* left bundle branch block, *LVH* left ventricular hypertrophy, *RBBB* right bundle branch block, *RVH* right ventricular hypertrophy, *PVC* premature ventricular contraction, *TWI* T-wave inversion

*Further evaluation if two or more ‘borderline’ ECG findings identified. Further evaluation required in the presence of any ‘abnormal’ ECG finding. No further evaluation required if ‘normal’ ECG findings are found in asymptomatic athletes that report no family history of inherited cardiac disease or SCD

## Athlete preference

There is no evidence that PPS deters young athletes from participating in competitive sports. On the contrary, screening to promote safe exercise is likely to raise awareness of cardiac disease, promote healthier life habits and achieve the most important goal of western health care organisations: a reduction in cardiovascular disease burden. A prospective, non-randomised controlled trial of 952 high school athletes demonstrated that athletes undergoing ECG screening were likely to more likely to be satisfied with their screening, feel safe during competition, support that all athletes should receive cardiac screening and state that the ECG had a positive impact on their training [36]. Individuals with false-positive screening tests were not found to report excessive anxiety after screening. Nevertheless, support mechanisms to assist athletes diagnosed with serious or potentially lethal cardiac disease should be considered an important part of PPS pathway.

## Cost of ECG screening

A central argument that has prevented the national legislation of ECG screening in young athletes in most countries relates to the financial implications of the practice [24]. Consequently, screening of athletes with ECG has largely been confined to athletes participating under the banner of financially endowed sporting organisations. The ECG itself is a relatively inexpensive test. Cost-effectiveness studies from the USA have reported the cost per athlete life saved to range from $44,000 to $204,000 [37, 38]. The reasons for such variation relate to inconsistencies in the methodology of the studies including variation in disease prevalence, variation in positive screening tests and differences in cost of investigations. The studies published to date are therefore not comparable and are unlikely to be applicable to many western countries where the cost of investigations is not as expensive as that in the USA. In a British study of nearly 5000 young athletes from 26 different sporting disciplines, modification of ECG interpretation criteria with the international recommendations was associated with significant reductions in the proportion of secondary investigations following screening and subsequently has been shown to reduce the cost of ECG screening by nearly 25% without compromising the ability to identify serious cardiac disease [35, 39]. These findings will be welcomed by sporting associations that mandate ECG screening in athletes and may allow less financially endowed organisations to afford ECG screening.

## Limitations of screening

### Natural history of the conditions being screened

The understanding of the natural history of the disease is an important feature for any screening program. In particular, a recognisable latent or early stage of the disease is essential. Given the heterogeneity of the diseases implicated in SCD and the low event rates in individuals with disease, it is arguable if their natural history can be fully appreciated. Commendable efforts have been made to risk stratify individuals identified with cardiac disease who are in the early stage of disease by virtue of being asymptomatic. For example, validated risk stratification tools exist to predict the prognosis of individuals diagnosed with HCM. Risk factors for SCD include unheralded syncope, family history of SCD, severe LVH (> 30 mm), sustained or non-sustained ventricular tachycardia, and attenuated blood pressure response to exercise [40]. Individuals exhibiting ≥ 1 of these five risk markers should be considered for prophylactic insertion of an implantable cardioverter defibrillator (ICD). The more recent risk stratification model proposed by the ESC HCM outcome group shows promise; however, the score was derived from a non-athletic cohort and therefore cannot readily be extrapolated to highly trained athletes diagnosed with disease [41].

Risk stratification for ion channel diseases implicated in SCD in young individuals may be achieved using the resting 12-lead ECG. A QT interval > 500 msec or the presence of the spontaneous type 1 Brugada pattern confer a higher risk of SCD to asymptomatic individuals diagnosed with LQTS and Brugada syndrome respectively [42]. Invasive electrophysiological evaluation of the asymptomatic individuals with the Wolf–Parkinson–White (WPW) ECG pattern may identify those at elevated risk for SCD. In particular, young patients with shortest pre-excited R-R interval (SPERRI) ≤ 250 msec in atrial fibrillation are at increased risk [43].

The majority of conditions implicated in SCD are not curative. Screening for these conditions has however been endorsed on the basis that there are acceptable interventions available to reduce the risk of SCD in young athletes identified with disease. Such interventions may include lifestyle changes, pharmacological therapy, radiofrequency ablation for accessory pathways or implantation of an ICD. Pharmacological therapy with beta blockers is an effective first-line treatment for LQTS and CPVT [42]. As with all individuals, an ICD is indicated for athletes considered to be a high risk of SCD and may be effective at reducing arrhythmogenic deaths in athletes harbouring structural or electrical cardiac disease [42, 44, 45].

### False negatives

The ECG cannot detect all disorders predisposing to SCD in athletes; for example, the ECG is normal and therefore unable to identify the majority (> 90%) of individuals with premature coronary artery disease or congenital coronary anomalies [24]. Furthermore, the ECG may be normal in 5–10% of athletes with HCM and in 25–30% of genetically affected individuals with LQTS [46, 47]. Similarly, the resting ECG is usually normal in individuals with CPVT [42].

### Variation in ECG interpretation

As with any subjective investigation, the effectiveness of the ECG is dependent on the individual interpretation of the test. A commonly cited criticism of the ECG as a screening test relate to the potential for variation in ECG interpretation especially in inexperienced hands. In a study assessing inter-observer agreement in ECG interpretation in athletes, cardiologists who do not routinely screen athletes (inexperienced cardiologists) were at least 40% more likely to categorise ECGs as abnormal compared to experienced cardiologists [23]. Additionally, inter-observer agreement rates for ECG interpretation among inexperienced cardiologists were inferior compared to their experienced counterparts. Whilst experience is undoubtedly useful, inter-observer agreement even amongst experienced cardiologists was moderate at best, highlighting the need for further training, and possibly accreditation to support cardiologists involved in screening young athletes. Recent small studies have demonstrated significant improvement in ECG interpretation in athletes following online training among inexperienced physicians and hold promise for the future [48, 49]. A study by Drezner et al. reported an overall improvement in the sensitivity of ECG interpretation from 89 to 94% and specificity from 70 to 91% before and after training with an online ECG interpretation tool based largely on the 2010 ESC recommendations [48]. Furthermore, the study reported high rates of agreement in ECG interpretation between cardiologists and other physicians (96% and 91%, *p* = 0.053) after training. Exeter et al. assessed ECG interpretation in two groups of physicians, one which received training with an online ECG interpretation tool (intervention group) and the other group who read the same ECG without the tool (control group) [49]. They demonstrated a reduction in false-positive ECG’s in the intervention group but no difference in sensitivity between the two groups.

## Conclusions

Prevention of SCD in young athletes remains the priority of the sports medicine community. With a better understanding of the physiological adaptations in athletes and the electrical consequences of such alterations, ECG criterion development to guide physicians has significantly improved the efficacy of ECG screening to acceptable levels of false-positive rates and high rates of sensitivity to identify athletes at risk of SCD. There is a compelling argument that these resources would be better off served in improving facilities, training and availability for cardiopulmonary resuscitation and AEDs. Although such practice is associated with a significant improvement in survival, it will generally capture events occurring in public places and sporting arenas [50–52]. A recent prospective study of young SCD in children and young adults in Australia and New Zealand reported that almost 40% cases of SCD occurred during sleep, and similar findings were also observed in a pathology series of athletic SCD in the UK [14, 53]. These findings suggest that early identification of individuals at risk through screening may be an important complementary strategy to reduce the overall burden of SCD in young athletes.
